# ENTERING MEDICARE ADVANTAGE FROM EMPLOYER-SPONSORED PLANS: CHANGE IN OUT-OF-POCKET COSTS AND UTILIZATION AT AGE 65

**DOI:** 10.1093/geroni/igac059.2895

**Published:** 2022-12-20

**Authors:** Alexandra Glynn Hames, Eric Roberts

**Affiliations:** University of Pittsburgh, Pittsburgh, Pennsylvania, United States; University of Pittsburgh, Pittsburgh, Pennsylvania, United States

## Abstract

An increasing proportion (nearly 40%) of older adults entering Medicare at age 65 are electing to enroll in private Medicare Advantage (MA) plans, which reflects more generous coverage relative to traditional Medicare and similarity to many features of employer sponsored plans. However, few studies have examined the impact of transitioning to MA from an employer-sponsored plan on out-of-pocket costs and health services utilization. We used longitudinal administrative data from Optum Insights between 2010 and 2021 to follow individuals from ages 62 through 67, spanning continuous enrollment in employer-sponsored insurance and MA. We employed an event-study design to estimate within-person changes in our outcomes after individuals entered to MA at age 65 (n=5,548), controlling for linear age trends. Relative to ages 62–64, MA enrollees at ages 66 and 67 had lower out-of-pocket spending on outpatient services (-$503/year, 95% CI: -$602, $404), emergency department visits (-$27/year, 95% CI: -$35, -$19), and prescription drugs (-$77/year, 95% CI: -$101, -$53). In contrast, utilization of outpatient services increased (1.4 visits/year, 95% CI: 0.8, 2.0), including physician evaluation and management visits (0.46 visits/year, 95% CI: 0.25, 0.68). After entering MA, individuals were more likely to have physician office visits with no out-of-pocket costs than at ages 62–64 (0.99 additional zero-cost visits/year, 95% CI: 0.81, 1.2). These results suggest MA may offer older adults more affordable care for routine and chronic conditions, compared to employer-sponsored insurance. These findings are timely as policymakers continue to deliberate proposals, such as expanding Medicare eligibility, to improve access to affordable insurance.

